# Supplementary material for: 4D printed hydrogel scaffold with swelling-stiffening properties and programmable deformation for minimally invasive implantation
Source: Nat Commun. 2024 Feb 21;15:1587. doi: 10.1038/s41467-024-45938-0 (PMC10881973; doi:10.1038/s41467-024-45938-0)
Supplement: Supplementary file 1 — Supplementary Information [file 41467_2024_45938_MOESM1_ESM.pdf]

Supplementary Information for

4D printed hydrogel scaffold with swelling-stiffening performance for programmable deformation and minimally invasive implantation

Bo Liu,[1,2] Hui Li,[1] Fengzhen Meng,[1] Ziyang Xu,[3] Liuzhi Hao,[1,4] Yuan Yao,[3] Hao Zhu,[1] Chenmin Wang,[1] Jun Wu,[5] Shaoquan Bian,[1] Willima W. Lu,[1,6] Wenguang Liu,[3]* Haobo Pan,[1]* Xiaoli Zhao[1,4]*

[1]Research Center for Human Tissue and Organs Degeneration, Institute of Biomedicine and Biotechnology, Shenzhen Institute of Advanced Technology, Chinese Academy of Sciences, Shenzhen 518055, China.

[2]Center for Health Science and Engineering, School of Health Sciences and Biomedical Engineering, Hebei University of Technology, Tianjin 300131, China.

[3]School of Materials Science and Engineering, Tianjin Key Laboratory of Composite and Functional Materials, Tianjin University, Tianjin 300350, China.

[4]University of Chinese Academy of Sciences, Beijing 100049, China.

[5]Shenzhen Key Laboratory for Innovative Technology in Orthopaedic Trauma, Department of Orthopaedics and Traumatology, The University of Hong Kong-Shenzhen Hospital, Shenzhen 518048, China.

[6]Department of Orthopaedics and Traumatology, Li Ka Shing Faculty of Medicine, The University of Hong Kong, Hong Kong 999077, China.

*Corresponding authors: wgliu@tju.edu.cn; hb.pan@siat.ac.cn; zhao.xl@siat.ac.cn

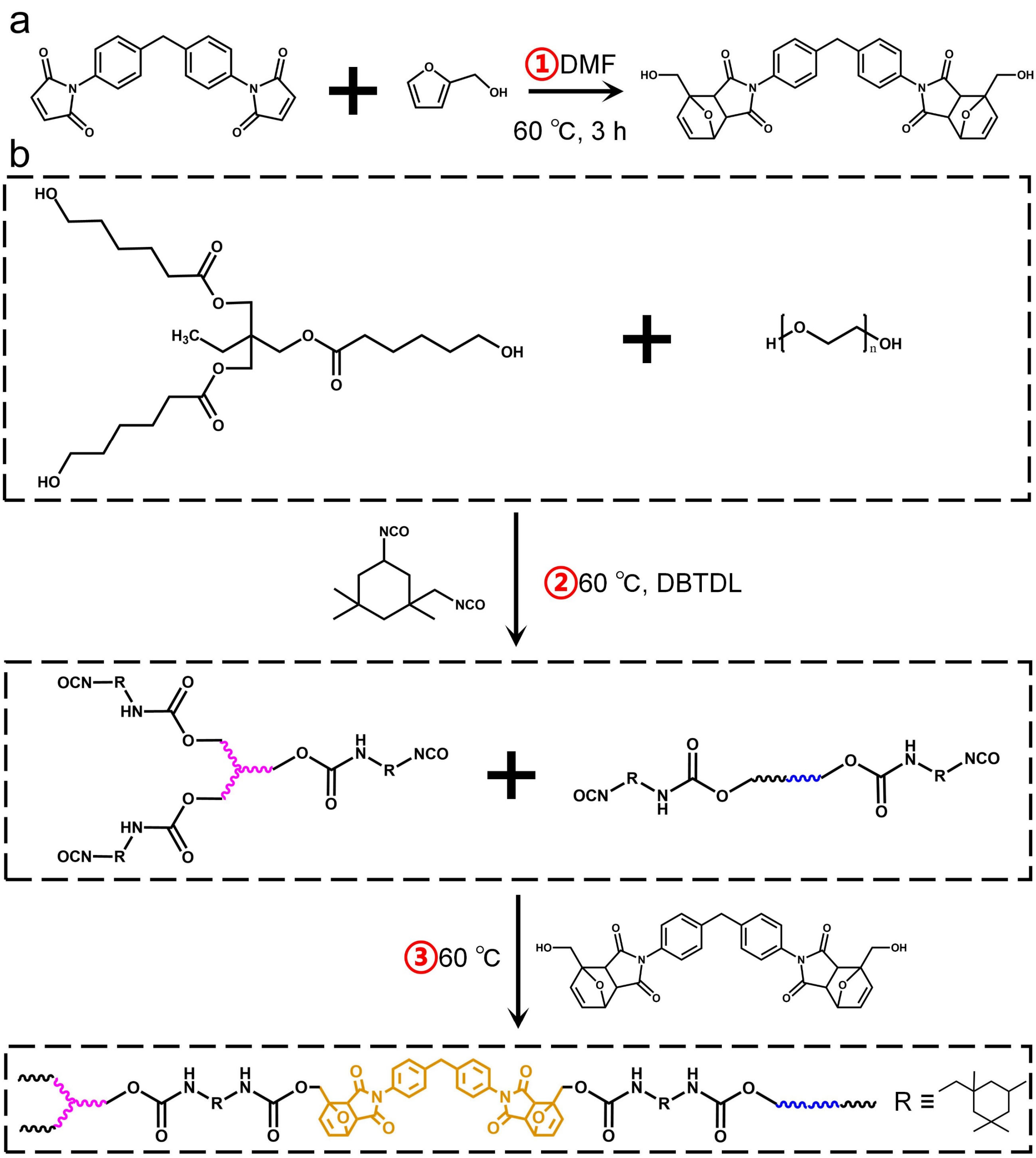

Supplementary Fig. 1

Synthesis of the DA-diol by DA reaction (a), the prepolymer by nucleophilic addition reaction, and the DTPU with DA-diol as the chain extender (b).

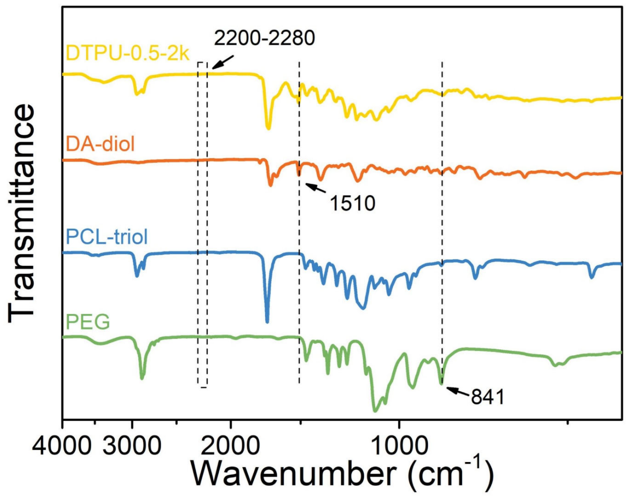

Supplementary Fig. 2

FTIR spectra of PEG, PCL-triol, DA-diol, and DTPU-0.5-2k. In the curves of DTPU-0.5-2k, an absorption peak was observed at 3330~3360 cm^{-1}, which was ascribed to the stretching vibration of -NH. A complete -NCO group consumption could be demonstrated by the absence of characteristic absorption at 2200-2280 cm^{-1} (N=C stretching of -NCO group). The absorption at 1510 cm^{-1} represented the stretching vibration of benzene ring, indicating the successful introduction of DA-diol. Meanwhile the rocking vibration peak at 841 cm^{-1} corresponding to CH_2 of PEG can reflect that PEG is present in DTPU-0.5-2k.

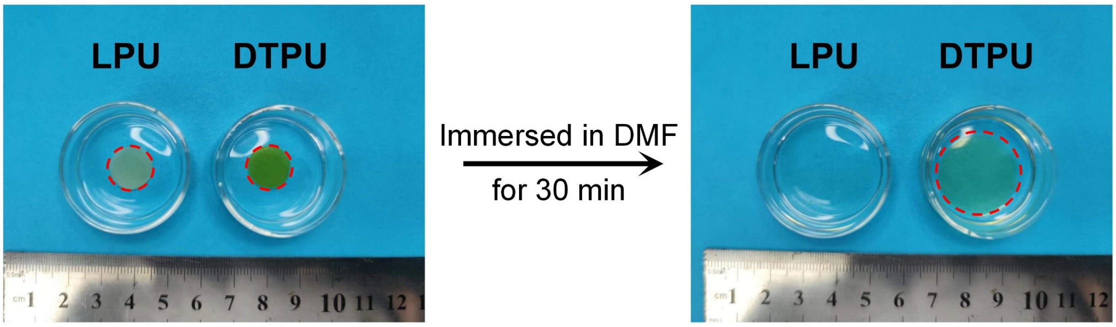

Supplementary Fig. 3

The solubility of LPU and DTPU in DMF. LPU and DTPU discs with a diameter of 1 cm and a thickness of 1 mm were immersed in DMF respectively. After standing for 30 min, LPU was completely dissolved, while DTPU swelled.

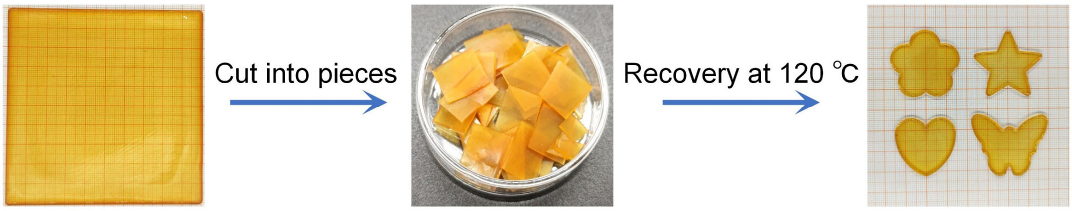

Supplementary Fig. 4

The recyclability of DTPU. The prepared DTPU film (10 cm × 10 cm × 0.5 mm) was cut into pieces and mixed with DMF. The mixture was heated at 120 °C for 20 min to ensure the complete dissolution of DTPU. Then, the solution was transferred into the Teflon mold and placed in a 60 °C oven for 2 days to remove DMF.

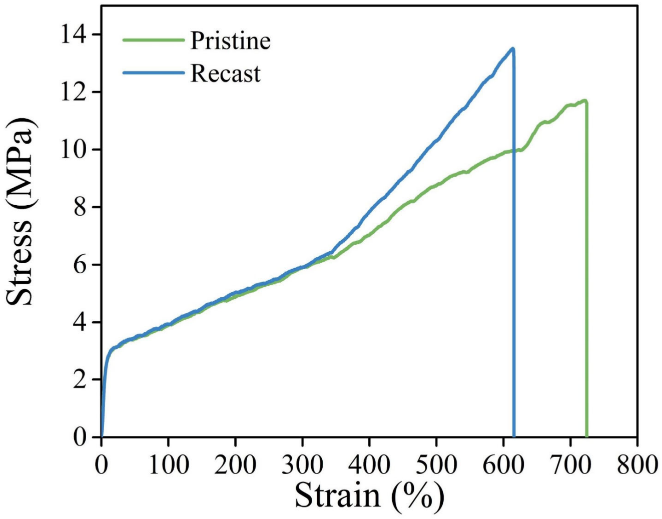

Supplementary Fig. 5

Tensile stress-strain curves for the pristine and recast DTPU-0.25-4k.

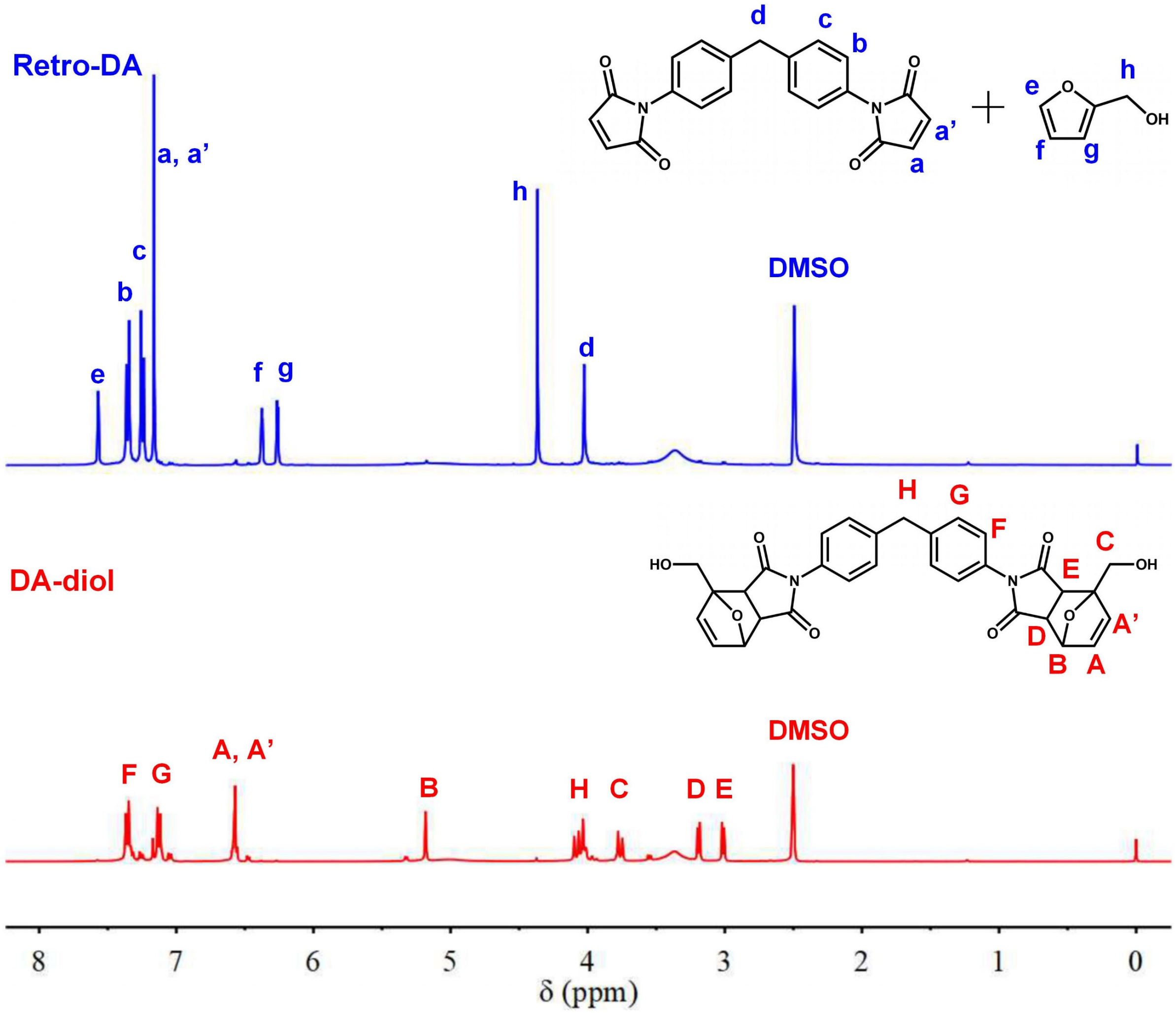

Supplementary Fig. 6

^{1}H NMR spectra of DA-diol and Retro-DA: Retro-DA is the DA-diol sample heated at 120 °C for 20 min.

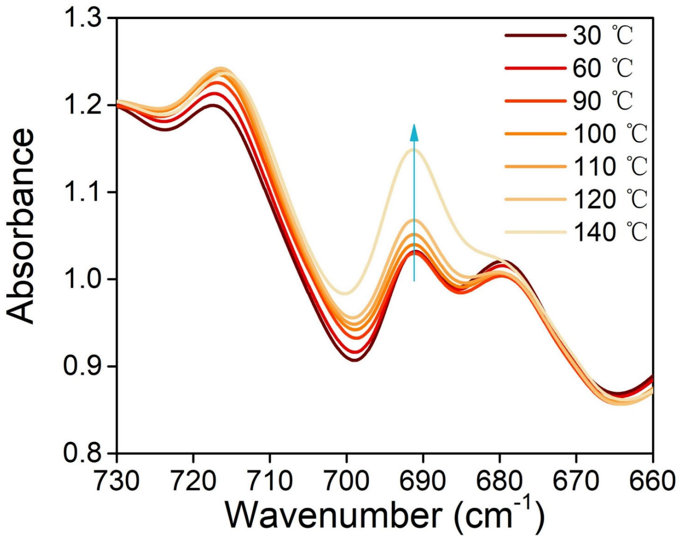

Supplementary Fig. 7

Temperature-dependent absorbance FTIR spectra of DTPU-0.5-2k film.

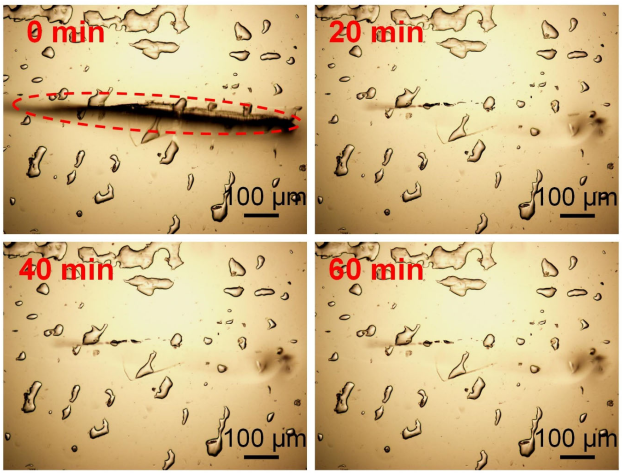

Supplementary Fig. 8

Optical microscope images of DTPU-0.25-4k damaged (0 min) and healed after different time under 60 °C (n = 3). The prepared DTPU film (30 mm × 10 mm × 0.5 mm) was attached to a slide by double-sided tape. And a scratch was created at the center of the film by using a scalpel. Subsequently, the slide carrying the film was placed in 60 °C oven for varying periods of time to examine the self-healing.

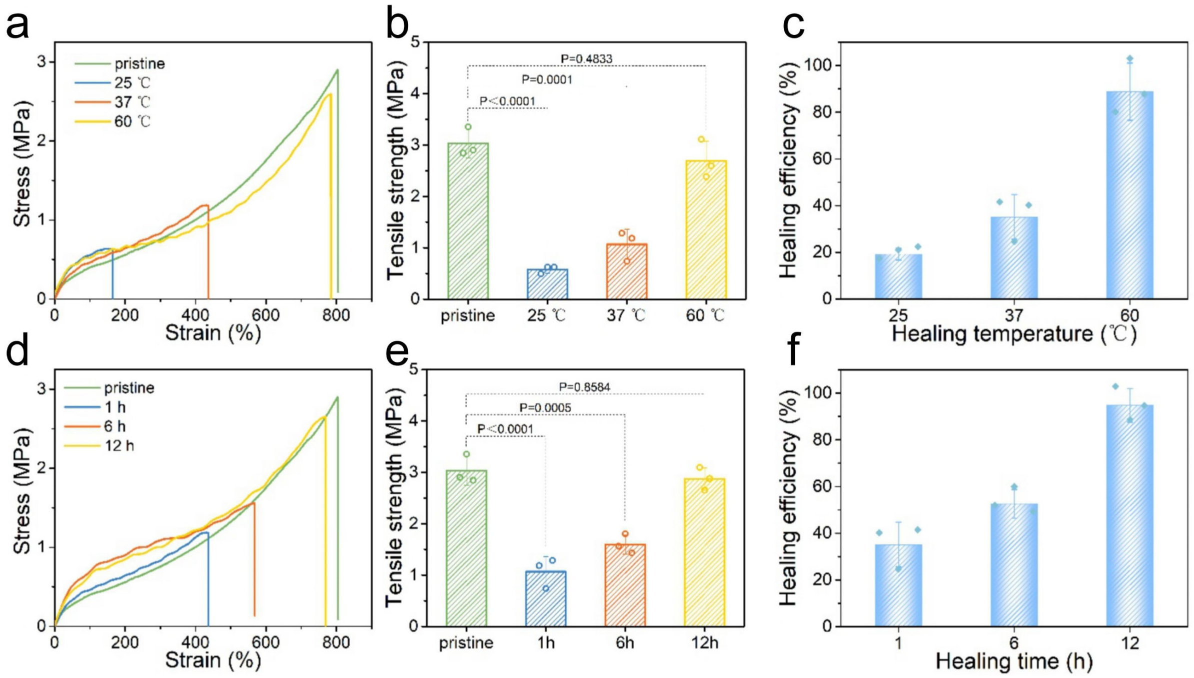

Supplementary Fig. 9

Tensile stress strain curves (a), tensile strength (b), and healing efficiency (c) of the self-healed DTPU-0.5-4k at various healing temperatures for 1 h. Tensile stress strain curves (d), tensile strength (e), and healing efficiency (f) of the self-healed DTPU-0.5-4k at 37 °C for various healing time. Values in (b), (c), (e), and (f) represent mean ± SD. One-way analysis of variance (ANOVA), Tukey's post hoc test. (n = 3).

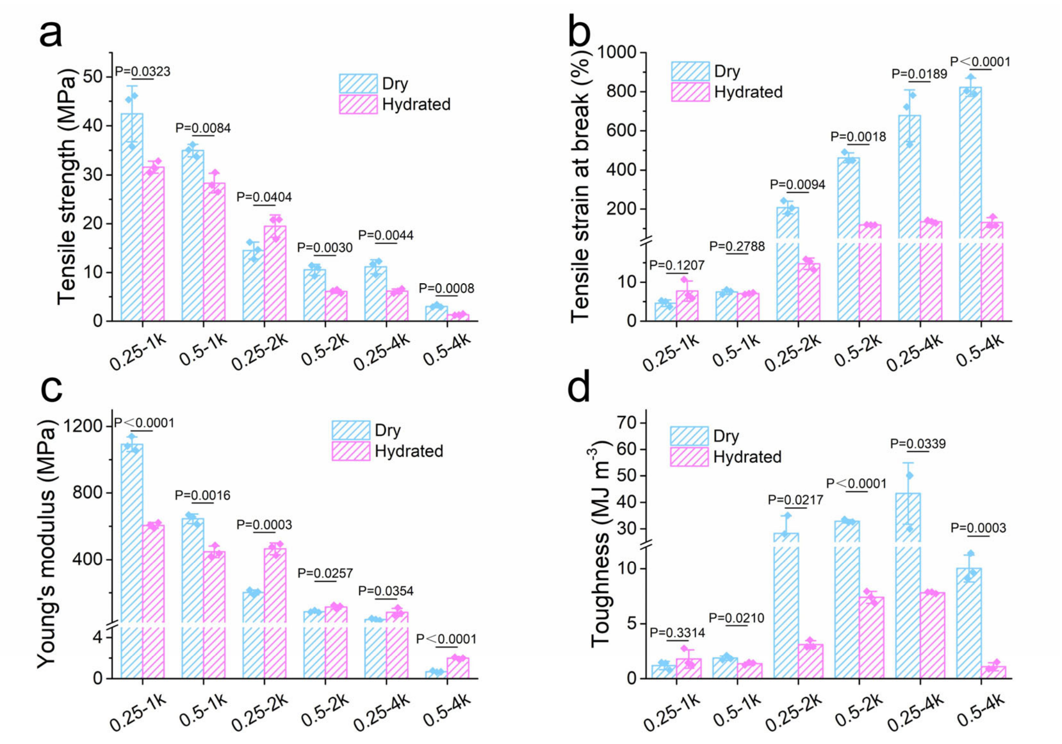

Supplementary Fig. 10

Tensile strength (a), tensile strain at break (b), Young's modulus (c), and toughness (d) of dry and hydrated DTPU with different PEG/PCL-triol and M_{PEG}. Values represent mean ± SD. Statistical analysis: two-tailed *t*-test. (n = 3). In (b), statistical analysis of DTPU-0.25-2k, DTPU-0.5-2k, and DTPU-0.25-4k groups were Welch-corrected. In (d), statistical analysis of DTPU-0.25-2k and DTPU-0.25-4k groups were Welch-corrected.

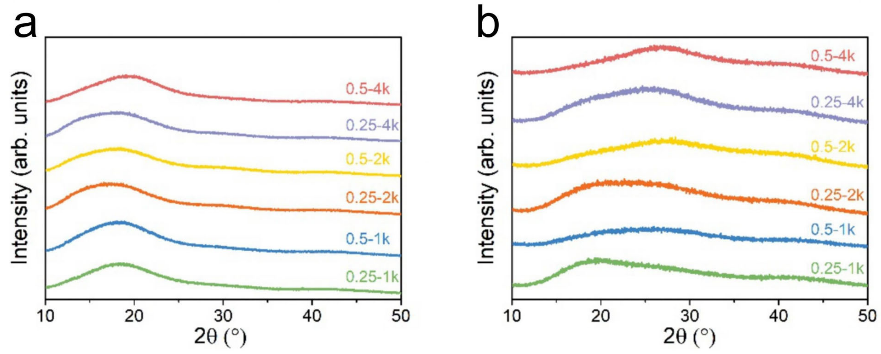

Supplementary Fig. 11

XRD for dry (a), and hydrated DTPU (b) with different PEG/PCL-triol and M_{PEG}.

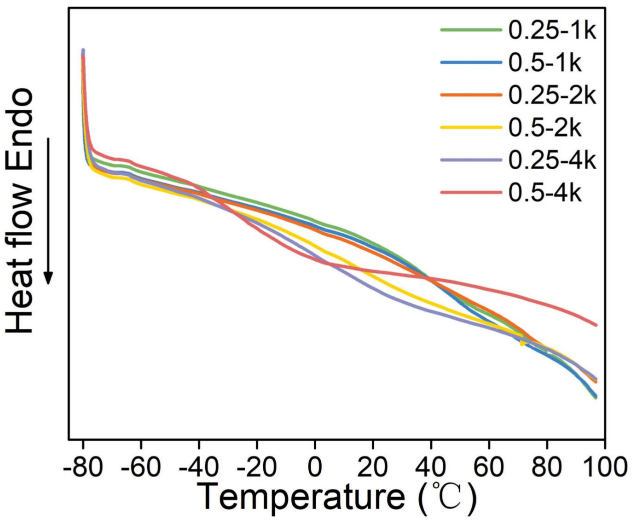

Supplementary Fig. 12

DSC for DTPU with different PEG/PCL-triol and M_{PEG}.

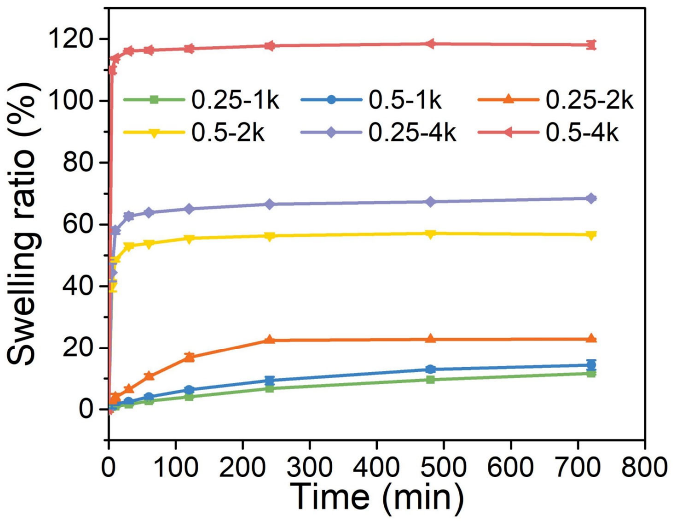

Supplementary Fig. 13

Swelling ratio over time curves of DTPU with different PEG/PCL-triol and M_{PEG}. Data are presented as mean values ± SD. (n = 3).

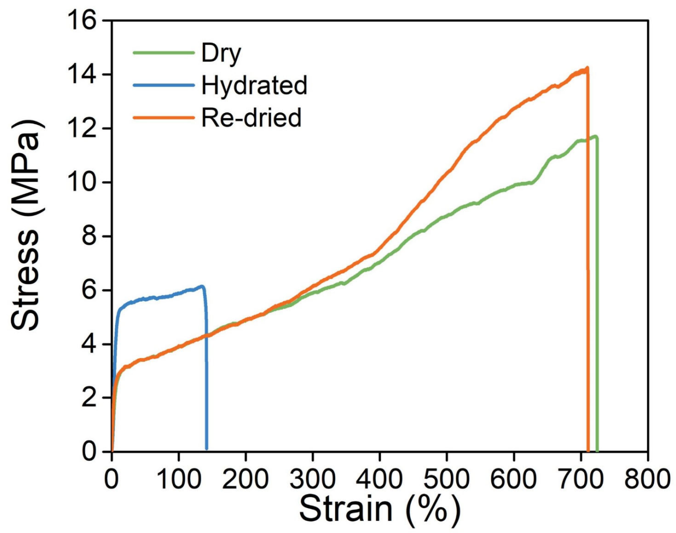

Supplementary Fig. 14
Tensile stress-strain curves for DTPU-0.25-4k in dry state, hydrated state, and re-dried state.

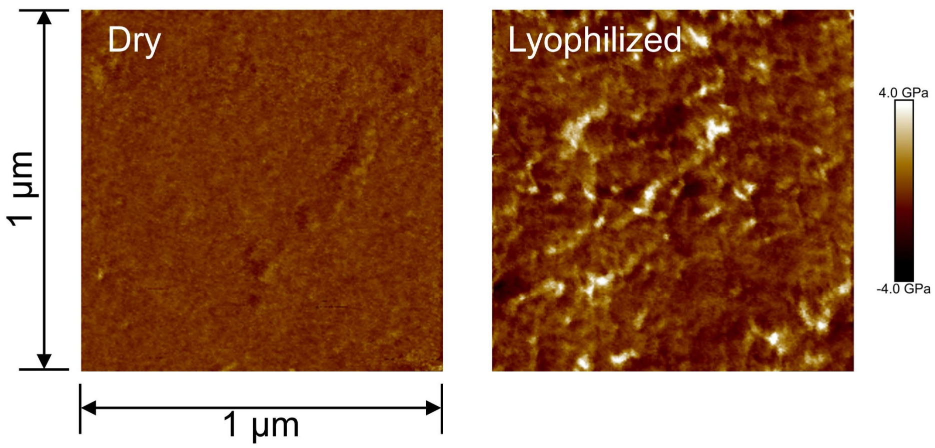

Supplementary Fig. 15
AFM images of DTPU-0.25-4k films in dry and lyophilized state (n = 3).

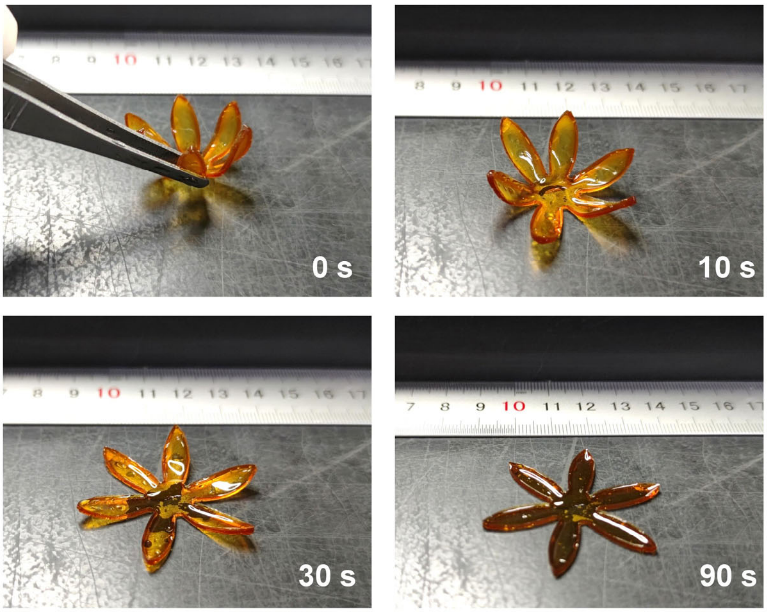

Supplementary Fig. 16

Shape recovery of DTPU-0.25-4k from temporary closed petals to initial 2D shape. The cast 2D flat petal was first fixed into a temporary 3D shape in cold water at 4 °C. Then, the petal with 3D temporary shape was placed on a 37 °C heating stage to allow shape recovery.

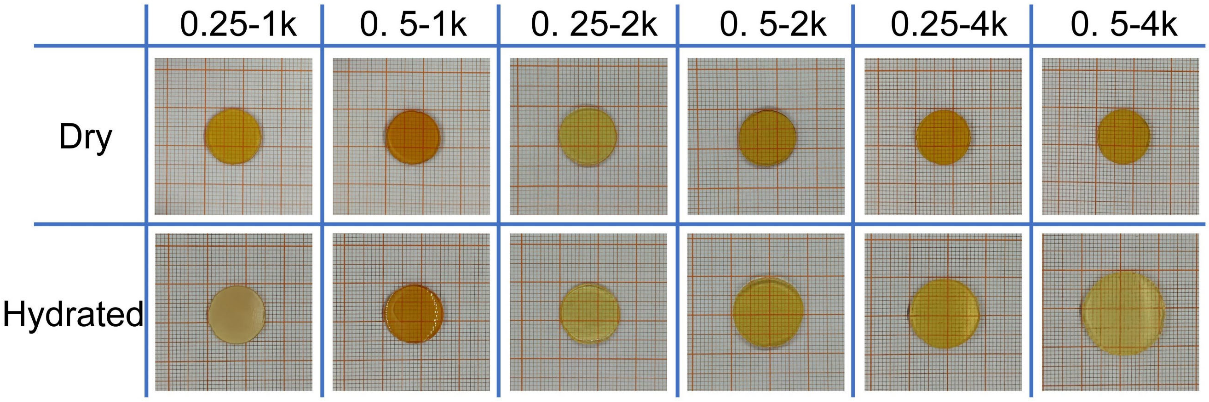

Supplementary Fig. 17

Digital images of volumetric change after hydration (n = 3). Dry DTPU films with the same shape were placed in deionized water at 37 °C for 2 days, and the deionized water was refreshed every 12 h. The volume of the film after swelling equilibrium was assessed.

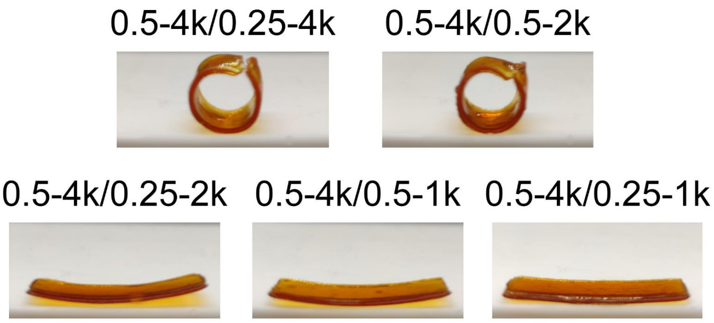

Supplementary Fig. 18

Bending angles of swelling mismatch structures after 10 min submersion in water. The stripe-shaped DTPU-0.5-4k (30 mm × 10 mm × 0.5 mm) was overlapped on different DTPU strips and placed in a 90 °C oven for 4 h to form a tightly bonded bilayer structure. Then, the bilayer was immersed in 37 °C deionized water, and the bending behavior was recorded after 10 min.

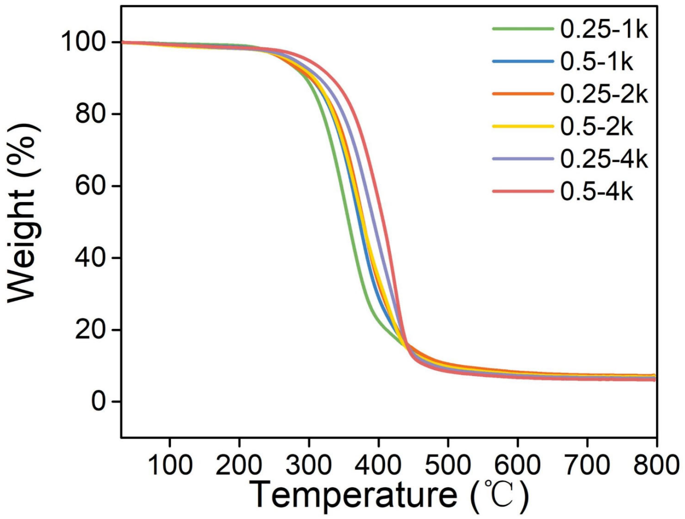

Supplementary Fig. 19

TGA for DTPU with different PEG/PCL-triol and M_{PEG}.

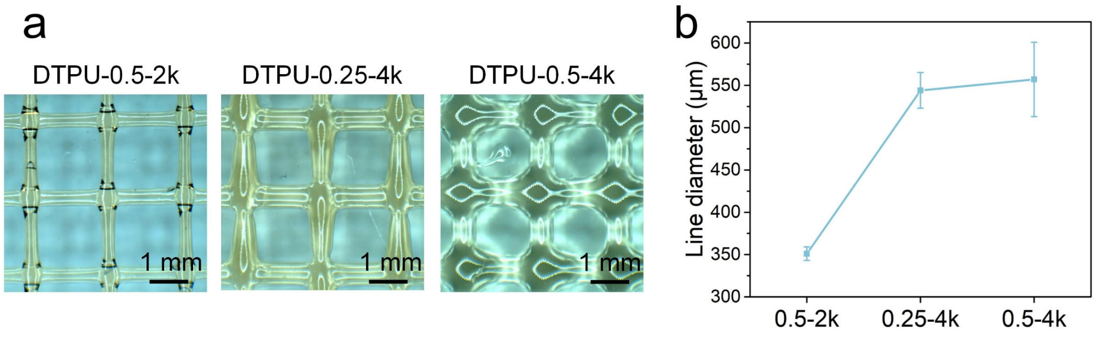

Supplementary Fig. 20

Amplified image of the printed grids (n = 3) (a). Shape fidelity of the DTPU (b). The grids were printed with 300 μm micronozzles and a line distance of 2 mm. Data in b are presented as mean values ± SD. (n = 3).

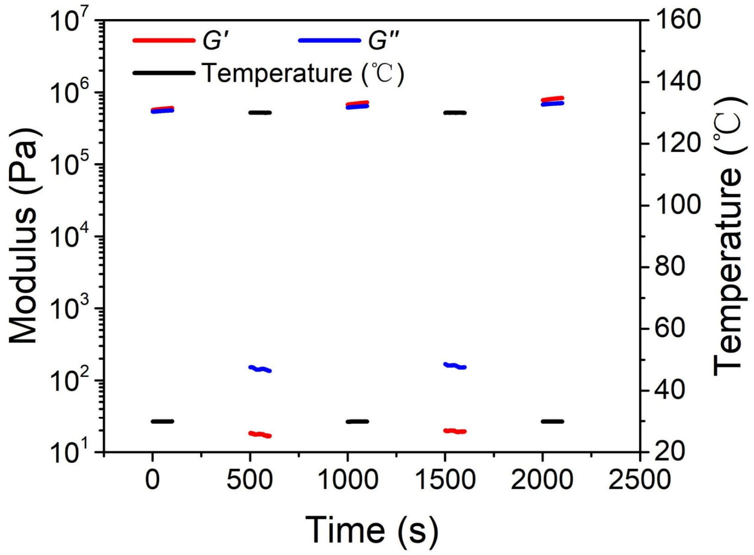

Supplementary Fig. 21

Oscillatory alternating temperature sweep of DTPU-0.5-4k.

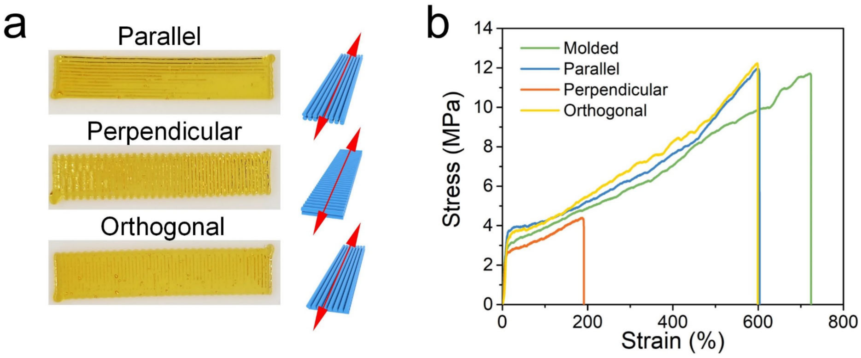

Supplementary Fig. 22

Top view of the printed tensile specimens with different printing direction, and a diagram of the direction of stretch (a). Tensile stress strain curves of DTPU-0.25-4k with different printing direction (b).

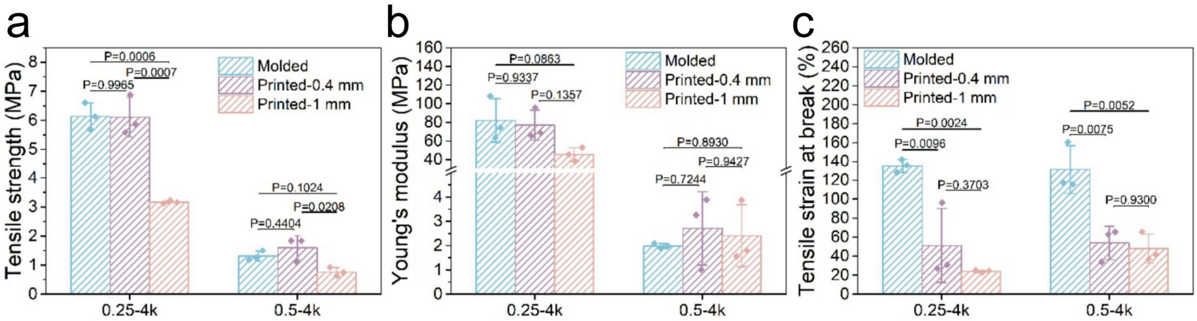

Supplementary Fig. 23

Tensile strength (a), Young's modulus (b), and tensile strain at break (c) of hydrated scaffolds constructed with different molding methods. Values represent mean ± SD. One-way analysis of variance (ANOVA), Tukey's post hoc test. (n = 3).

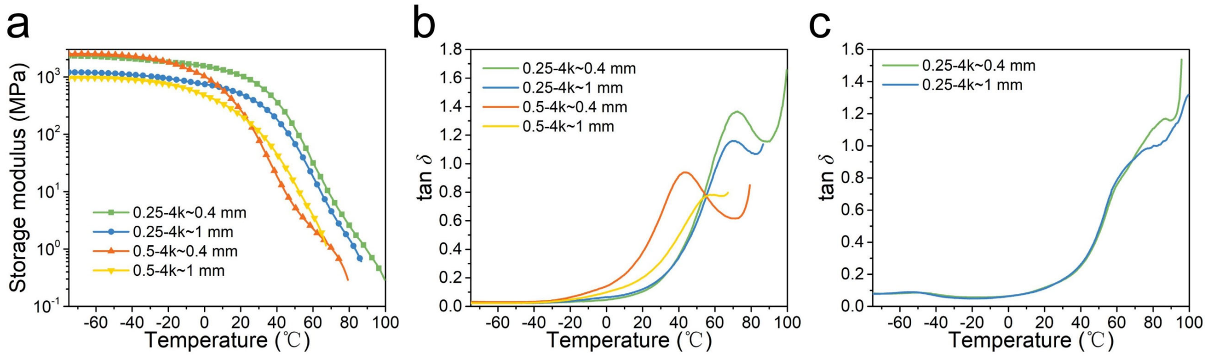

Supplementary Fig. 24

Storage modulus-temperature curve (a), and tan δ-temperature curve (b) of dry flat scaffolds printed using DTPU-0.25-4k or DTPU-0.5-4k with different line distance. Tan δ-temperature curve of hydrated DTPU-0.25-4k scaffolds printed with different line distance (c).

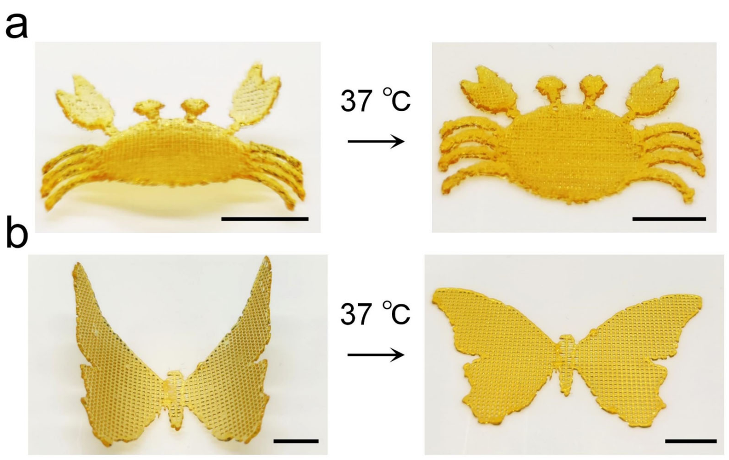

Supplementary Fig. 25

Shape recovery of a crab (a) and a butterfly (b) printed with DTPU-0.25-4k from temporary vivid 3D shapes to initial 2D shapes. Scale bars:10 mm.

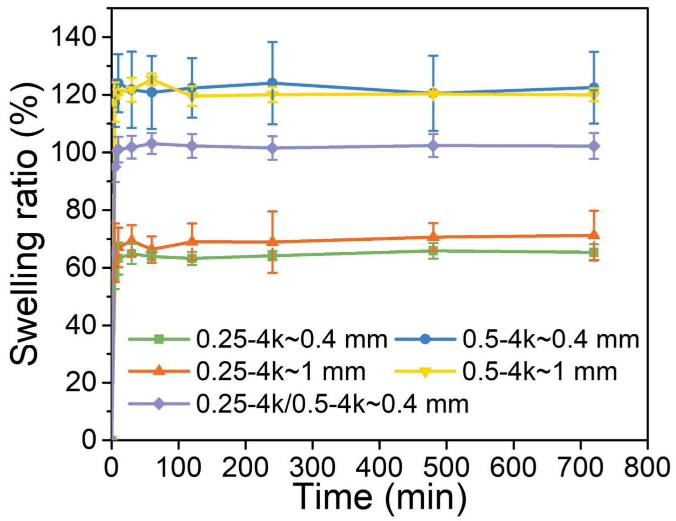

Supplementary Fig. 26

Swelling ratio over time curves of scaffolds printed using DTPU-0.25-4k and/or DTPU-0.5-4k with different line distance. Data are presented as mean values ± SD. (n = 3).

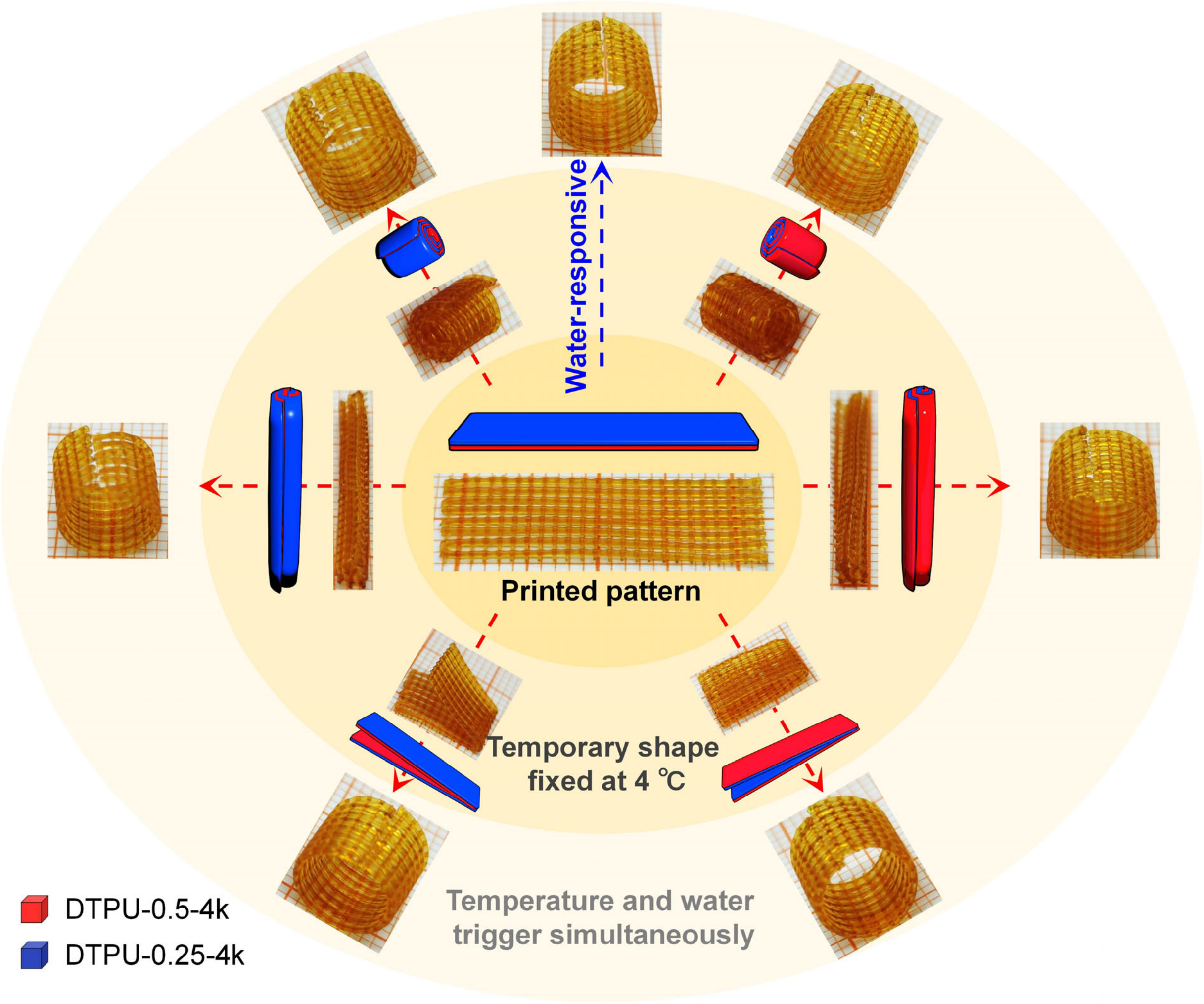

Supplementary Fig. 27

Images of final 3D shapes formed in response to simultaneous temperature and water under different fixation patterns. The inner layer shows the initial shape of the 2D flat pattern and the corresponding model. The middle layer exhibits the temporary shape after being rolled or folded in different ways and then fixed at 4 °C. The corresponding model demonstrates the specific fixation method. The outer layer shows the final shape of the corresponding temporary shape in response to simultaneous stimulation of temperature and water (37 °C water).

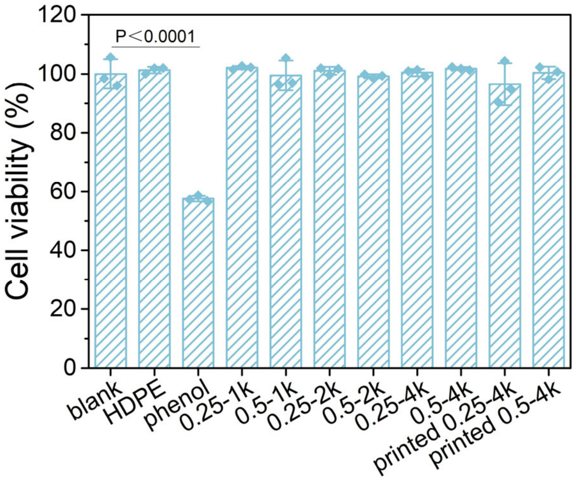

Supplementary Fig. 28

Cytotoxicity analysis of DTPUs casted or printed with different PEG/PCL-triol and M_{PEG}. Values represent mean ± SD. One-way analysis of variance (ANOVA), Tukey's post hoc test. (n = 3).
