# Supplementary material for: 4D printed hydrogel scaffold with swelling-stiffening properties and programmable deformation for minimally invasive implantation
Source: Nat Commun. 2024 Feb 21;15:1587. doi: 10.1038/s41467-024-45938-0 (PMC10881973; doi:10.1038/s41467-024-45938-0)
Supplement: Supplementary file 3 — Description of Additional Supplementary Files [file 41467_2024_45938_MOESM3_ESM.pdf]

Description of Additional Supplementary Files

File Name: Supplementary Movie 1
Description: Capacity of the DTPU-0.25-4k to resist deformation at different temperatures.

File Name: Supplementary Movie 2
Description: Thermal-induced shape memory and recovery processes of DTPU-0.25-4k petal.

File Name: Supplementary Movie 3
Description: *In vitro* demonstration of 4D printed scaffold for transcatheter delivery and multi-endogenous stimuli responsiveness.

File Name: Supplementary Movie 4
Description: *In vivo* demonstration of 4D printed scaffold for transcatheter delivery and multi-endogenous stimuli responsiveness.
