# Supplementary material for: 4D printed hydrogel scaffold with swelling-stiffening properties and programmable deformation for minimally invasive implantation
Source: Nat Commun. 2024 Feb 21;15:1587. doi: 10.1038/s41467-024-45938-0 (PMC10881973; doi:10.1038/s41467-024-45938-0)
Supplement: Supplementary file 8 — Reporting Summary [file 41467_2024_45938_MOESM8_ESM.pdf]

nature portfolio

Corresponding author(s): Wenguang Liu; Haobo Pan; Xiaoli Zhao

Last updated by author(s): Jan 30, 2024

Reporting Summary

Nature Portfolio wishes to improve the reproducibility of the work that we publish. This form provides structure for consistency and transparency in reporting. For further information on Nature Portfolio policies, see our Editorial Policies and the Editorial Policy Checklist.

Statistics

For all statistical analyses, confirm that the following items are present in the figure legend, table legend, main text, or Methods section.

n/a	Confirmed	
☐	☒	The exact sample size (*n*) for each experimental group/condition, given as a discrete number and unit of measurement
☐	☒	A statement on whether measurements were taken from distinct samples or whether the same sample was measured repeatedly
☐	☒	The statistical test(s) used AND whether they are one- or two-sided<br>*Only common tests should be described solely by name; describe more complex techniques in the Methods section.*
☒	☐	A description of all covariates tested
☒	☐	A description of any assumptions or corrections, such as tests of normality and adjustment for multiple comparisons
☐	☒	A full description of the statistical parameters including central tendency (e.g. means) or other basic estimates (e.g. regression coefficient) AND variation (e.g. standard deviation) or associated estimates of uncertainty (e.g. confidence intervals)
☐	☒	For null hypothesis testing, the test statistic (e.g. *F*, *t*, *r*) with confidence intervals, effect sizes, degrees of freedom and *P* value noted<br>*Give P values as exact values whenever suitable.*
☒	☐	For Bayesian analysis, information on the choice of priors and Markov chain Monte Carlo settings
☒	☐	For hierarchical and complex designs, identification of the appropriate level for tests and full reporting of outcomes
☒	☐	Estimates of effect sizes (e.g. Cohen's *d*, Pearson's *r*), indicating how they were calculated

Our web collection on statistics for biologists contains articles on many of the points above.

Software and code

Policy information about availability of computer code

Data collection	Data were collected with softwares as following: Care_Test_Fatigue (v1.6.2.0) and Bluehill 3 for tensile test, Nanoscope 10.0 for AFM analysis, QSeries (Q800-2570 - DMA Q800@Mfg-dma) for DMA analysis, Q2000-3186 for DSC analysis, EVA (v4.2.1) and TOPAS (v5) for XRD analysis, Diffrac.SAXS for SAXS analysis, OneAttension (Version 3.2) for water contact angle analysis, Anton Paar RheoCompass 1.24 for rheological analysis, OPUS 8.2.28 for FTIR analysis, and Topspin3,2 for NMR analysis.
Data analysis	OriginPro 2016, MestReNova14, ChemDraw 19.1, Grapher 12, Nanoscope analysis 3.0, GraphPad Prism9.5.

For manuscripts utilizing custom algorithms or software that are central to the research but not yet described in published literature, software must be made available to editors and reviewers. We strongly encourage code deposition in a community repository (e.g. GitHub). See the Nature Portfolio guidelines for submitting code & software for further information.

Data

Policy information about availability of data

All manuscripts must include a data availability statement. This statement should provide the following information, where applicable:

- Accession codes, unique identifiers, or web links for publicly available datasets
- A description of any restrictions on data availability
- For clinical datasets or third party data, please ensure that the statement adheres to our policy

The data generated in this study are provided in the Supplementary Information/Source Data file. The full image dataset is available from the corresponding author

on request. Source data are provided with this paper.

Research involving human participants, their data, or biological material

Policy information about studies with human participants or human data. See also policy information about sex, gender (identity/presentation), and sexual orientation and race, ethnicity and racism.

Reporting on sex and gender	N/A
Reporting on race, ethnicity, or other socially relevant groupings	N/A
Population characteristics	N/A
Recruitment	N/A
Ethics oversight	N/A

Note that full information on the approval of the study protocol must also be provided in the manuscript.

Field-specific reporting

Please select the one below that is the best fit for your research. If you are not sure, read the appropriate sections before making your selection.

[x] Life sciences [ ] Behavioural & social sciences [ ] Ecological, evolutionary & environmental sciences

For a reference copy of the document with all sections, see nature.com/documents/nr-reporting-summary-flat.pdf

Life sciences study design

All studies must disclose on these points even when the disclosure is negative.

Sample size	The samples used for testing in this manuscript were prepared according to the procedure outlined in the Methods section. Each sample was replicated a minimum of three times, guaranteeing sufficient data for accurate statistical analysis.Three sets of parallel data are the minimum requirements to eliminate and reduce errors.
Data exclusions	No data were excluded from the analyses.
Replication	All experiments were conducted using independent replicates, as shown in the legend.
Randomization	All experiment sample/animal in this manuscript were allocated randomly.
Blinding	The investigators were blinded to group allocation during data collection and analysis.

Reporting for specific materials, systems and methods

We require information from authors about some types of materials, experimental systems and methods used in many studies. Here, indicate whether each material, system or method listed is relevant to your study. If you are not sure if a list item applies to your research, read the appropriate section before selecting a response.

Materials & experimental systems

n/a	Involved in the study	
[x]	[ ]	Antibodies
[ ]	[x]	Eukaryotic cell lines
[x]	[ ]	Palaeontology and archaeology
[ ]	[x]	Animals and other organisms
[x]	[ ]	Clinical data
[x]	[ ]	Dual use research of concern
[x]	[ ]	Plants

Methods

n/a	Involved in the study	
[x]	[ ]	ChIP-seq
[x]	[ ]	Flow cytometry
[x]	[ ]	MRI-based neuroimaging

Eukaryotic cell lines

Policy information about cell lines and Sex and Gender in Research

Cell line source(s)	The L929 cells used in this study were commercially obtained from iCell Bioscience Inc (Shanghai).
Authentication	L929 cells were identified by STR.
Mycoplasma contamination	Cell lines used in this study were not contaminated with mycoplasma.
Commonly misidentified lines (See ICLAC register)	No commonly misidentified lines were used in the study.

Animals and other research organisms

Policy information about studies involving animals; ARRIVE guidelines recommended for reporting animal research, and Sex and Gender in Research

Laboratory animals	Sprague-Dawley rats weighing 190-210g (6-week-old) were obtained from Zhuhai BesTest Bio-Tech Co,.Ltd. and Beijing HFK BIOSCIENCE Co., Ltd., and used to subcutaneous implant polymer scaffolds.
Wild animals	No wild animals were used in the study.
Reporting on sex	Sex was not considered in this study design.
Field-collected samples	No field collected samples were used in this study.
Ethics oversight	In vivo experimental procedures were performed following the the Guide for the Care and Use of Laboratory Animals 8th edition (National Institutes of Health, Nat. Acad. Press, 2011) and Tianjin Medical Experimental Animal Care, with project approval from the Institutional Animal Care and Use Committee of the Shenzhen Institutes of Advanced Technology, Chinese Academy of Sciences (approval numbers: SIAT-IACUC-190328-YYS-ZXL-A0727), and the Institutional Animal Care and Use Committee of Yi Shengyuan Gene Technology (Tianjin) Co., Ltd. (protocol number: YSY-DWLL-2023252).

Note that full information on the approval of the study protocol must also be provided in the manuscript.
